# 9α-Hy­droxy-12-{[4-(4-meth­oxy­phen­yl)piperazin-1-yl]meth­yl}-4,8-dimethyl-3,14-dioxatricyclo­[9.3.0.0^2,4^]tetra­dec-7-en-13-one

**DOI:** 10.1107/S1600536812003662

**Published:** 2012-02-04

**Authors:** Mohamed Moumou, Ahmed Benharref, Jean-Claude Daran, Fouad Mellouki, Moha Berraho

**Affiliations:** a Laboratoire de Chimie Biomoléculaire, Substances Naturelles et Réactivité, URAC 16, Faculté des Sciences Semlalia, BP 2390, Boulevard My Abdellah, 40000 Marrakech, Morocco; b Laboratoire de Chimie de Coordination, 205 route de Narbonne, 31077 Toulouse Cedex 04, France; c Laboratoire de Chimie Bioorganique et Analytique, URAC 22, BP 146, FSTM, Université Hassan II, Mohammedia-Casablanca 20810 Mohammedia, Morocco

## Abstract

The title compound, C_26_H_36_N_2_O_5_, was synthesized from 9α-hy­droxy­parthenolide (9α-hy­droxy-4,8-dimethyl-12-methyl­ene-3,14-dioxatricyclo­[9.3.0.0^2,4^]tetra­dec-7-en-13-one), wich was isolated from the chloro­form extract of the aerial parts of *Anvillea radiata*. The mol­ecule is built up from fused five- and ten-membered rings with the meth­oxy­phenyl­piperazine group as a substituent. The ten-membered ring adopts an approximate chair–chair conformation, while the piperazine ring displays a chair conformation and the five-membered ring a flattened envelope conformation; the C(H)—C—C(H) atoms representing the flap lie out of the mean plane through the remaining four atoms by 0.343 (3) Å. The dihedral angle between the mean planes of the ten-membered ring and the lactone ring is 18.12 (14)°. An intra­molecular O—H⋯N hydrogen bond occurs. The crystal structure features weak C—H⋯O inter­actions.

## Related literature

For background to the medicinal uses of the plant *Anvillea radiata*, see: Abdel Sattar *et al.* (1996 ▶); Bellakhdar (1997 ▶); El Hassany *et al.* (2004 ▶); Qureshi *et al.* (1990 ▶). For ring-puckering parameters, see: Cremer & Pople (1975 ▶).
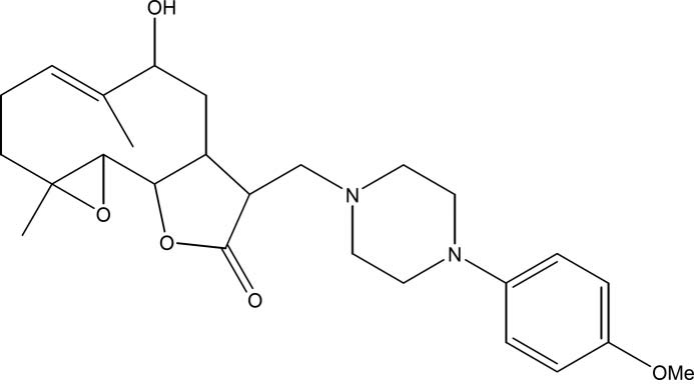


## Experimental

### 

#### Crystal data

C_26_H_36_N_2_O_5_
*M* *_r_* = 456.57
Orthorhombic, 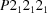

*a* = 6.7066 (7) Å
*b* = 11.9033 (11) Å
*c* = 30.322 (4) Å
*V* = 2420.6 (4) Å^3^
*Z* = 4
Mo *K*α radiation
μ = 0.09 mm^−1^
*T* = 180 K
0.33 × 0.17 × 0.04 mm

#### Data collection

Agilent Xcalibur Sapphire1 (long nozzle) diffractometer
Absorption correction: multi-scan (*CrysAlis PRO*; Agilent, 2010 ▶) *T*_min_ = 0.732, *T*_max_ = 1.000
14543 measured reflections
4925 independent reflections
3663 reflections with *I* > 2σ(*I*)
*R*_int_ = 0.055

#### Refinement

*R*[*F* ^2^ > 2σ(*F* ^2^)] = 0.055
*wR*(*F* ^2^) = 0.130
*S* = 1.04
4925 reflections
303 parameters
H-atom parameters constrained
Δρ_max_ = 0.25 e Å^−3^
Δρ_min_ = −0.23 e Å^−3^

### 

Data collection: *CrysAlis PRO* (Agilent, 2010 ▶); cell refinement: *CrysAlis PRO*; data reduction: *CrysAlis PRO*; program(s) used to solve structure: *SHELXS97* (Sheldrick, 2008 ▶); program(s) used to refine structure: *SHELXL97* (Sheldrick, 2008 ▶); molecular graphics: *ORTEP-3 for Windows* (Farrugia, 1997 ▶)and *PLATON* (Spek, 2009 ▶); software used to prepare material for publication: *WinGX* (Farrugia, 1999 ▶).

## Supplementary Material

Crystal structure: contains datablock(s) I, global. DOI: 10.1107/S1600536812003662/ds2172sup1.cif

Structure factors: contains datablock(s) I. DOI: 10.1107/S1600536812003662/ds2172Isup2.hkl

Supplementary material file. DOI: 10.1107/S1600536812003662/ds2172Isup3.cml

Additional supplementary materials:  crystallographic information; 3D view; checkCIF report

## Figures and Tables

**Table 1:** Hydrogen-bond geometry (Å, °)

*D*—H⋯*A*	*D*—H	H⋯*A*	*D*⋯*A*	*D*—H⋯*A*
O4—H4⋯N1	0.84	2.14	2.977 (4)	170
C2—H2⋯O12^i^	1.00	2.42	3.225 (4)	137
C5—H5*B*⋯O3^ii^	0.99	2.45	3.310 (4)	145
C7—H7⋯O14^iii^	0.95	2.50	3.198 (4)	130
C15—H15*A*⋯O12^i^	0.99	2.57	3.413 (4)	143
C15—H15*A*⋯O14^i^	0.99	2.50	3.469 (4)	165

Symmetry codes: (i) 
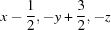
; (ii) 
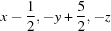
; (iii) 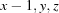.

**Fig. 1.**
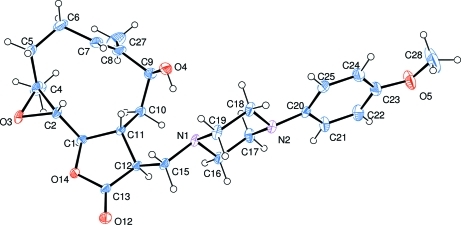
Molecular structure of the title compound with displacement ellipsoids drawn at the 30% probability level. H atoms are represented as small spheres of arbitrary radii.

**Fig. 2.**
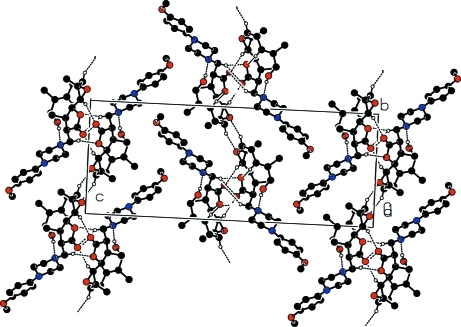
Partial packing view showing the C—H···O hydrogen bonds as dashed lines. H atoms not involved in hydrogen bonding have been omitted for clarity.

## supplementary crystallographic information

### Comment

Our work lies within the framework of the valorization of medicinals plants and concerning the *Anvillea radiata* wich is a plant that grows in northern Africa and particularly found in the two Maghreb countries, Morocco and Algeria. This plant is used in traditional local medicine for the treatment of dysentery, gastric-intestinal disorders (Bellakhdar, 1997), hypoglycemic activity (Qureshi *et al.*, 1990), and has been reported to have antitumoral activity (Abdel Sattar *et al.*, 1996). In our study of different Moroccan endemic plants, we have demonstrated that the aerial parts of *Anvillea radiata* could be used as a renewable source of 9-hydroxyparthenolide (El Hassany *et al.*, 2004). In order to prepare products with high added value that can be used in pharmacology and cosmetics industry, we studied the chemical reactivity of this major constituent of *Anvillea radiata*. Thus, treatment of this sesquiterpene lactone by an equivalent amount of 1-(4-methoxyphenylpiperazine) in ethanol led to the title compound with a yield of 78%. The crystal structure of (I) is reported herein. The molecule contains a fused ring system and methoxyphenylpiperazine group as a substituent to a lactone ring. The molecular structure of (I), Fig. 1, shows the lactone ring to adopt an envelope conformation, as indicated by Cremer & Pople (1975) puckering parameters Q = 0.216 (3) Å and φ = 69.7 (8)°. The atom C11 deviate from the mean plane through other four atoms in the ring by 0.343 (2) Å. The ten-membered ring displays an approximate chair–chair conformation, while the piperazine ring has a perfect chair conformation with QT = 0.557 (3) Å, θ = 3.4 (3)° and φ2 = 33 (6)°. In the crystal structure, the molecules are linked by C—H···O intermolecular hydrogen bonds into chains along the *b* axis (Table 1, Fig.2). In addition an intramolecular O—H···N hydrogen bond is also observed.

### Experimental

The mixture of 9α-hydroxyparthenolide (1 g, (3.78 mmol) and one equivalent of 1-(4-methoxyphenylpipirazine) in EtOH (30 ml) was stirred for one night at room temperature. The next day the reaction was stopped by adding water (20 ml) and extracted three times with ethyl acetate (3 × 30 ml). The combined organic layers were dried over anhydrous MgSO_4_, filtered and concentrated under vacuum to give 1.34 g (2.94 mmol) of the title compound, which was recrystallized in ethyl acetate.

### Refinement

All H atoms were fixed geometrically and treated as riding with C—H = 0.96 Å (methyl), 0.97 Å (methylene), 0.98 Å (methine) with *U*_iso_(H) = 1.2*U*_eq_(methylene, methine) or *U*_iso_(H) = 1.5*U*_eq_(methyl, OH). In the absence of significant anomalous scattering, the absolute configuration could not be reliably determined and any references to the Flack parameter were removed.

### Crystal data

**Table:**

C_26_H_36_N_2_O_5_	*F*(000) = 984
*M**_r_* = 456.57	*D*_x_ = 1.253 Mg m^−^^3^
Orthorhombic, *P*2_1_2_1_2_1_	Mo *K*α radiation, λ = 0.71073 Å
Hall symbol: P 2ac 2ab	Cell parameters from 14543 reflections
*a* = 6.7066 (7) Å	θ = 3.1–26.4°
*b* = 11.9033 (11) Å	µ = 0.09 mm^−^^1^
*c* = 30.322 (4) Å	*T* = 180 K
*V* = 2420.6 (4) Å^3^	Platelet, colourless
*Z* = 4	0.33 × 0.17 × 0.04 mm

### Data collection

**Table:**

Agilent Xcalibur Sapphire1 (long nozzle) diffractometer	4925 independent reflections
Radiation source: fine-focus sealed tube	3663 reflections with *I* > 2σ(*I*)
Graphite monochromator	*R*_int_ = 0.055
Detector resolution: 8.2632 pixels mm^-1^	θ_max_ = 26.4°, θ_min_ = 3.1°
ω scans	*h* = −8→8
Absorption correction: multi-scan (*CrysAlis PRO*; Agilent, 2010)	*k* = −14→14
*T*_min_ = 0.732, *T*_max_ = 1.000	*l* = −37→37
14543 measured reflections	

### Refinement

**Table:**

Refinement on *F*^2^	Secondary atom site location: difference Fourier map
Least-squares matrix: full	Hydrogen site location: inferred from neighbouring sites
*R*[*F*^2^ > 2σ(*F*^2^)] = 0.055	H-atom parameters constrained
*wR*(*F*^2^) = 0.130	*w* = 1/[σ^2^(*F*_o_^2^) + (0.0567*P*)^2^] where *P* = (*F*_o_^2^ + 2*F*_c_^2^)/3
*S* = 1.04	(Δ/σ)_max_ = 0.001
4925 reflections	Δρ_max_ = 0.25 e Å^−^^3^
303 parameters	Δρ_min_ = −0.23 e Å^−^^3^
0 restraints	Extinction correction: *SHELXL97* (Sheldrick, 2008), Fc^*^=kFc[1+0.001xFc^2^λ^3^/sin(2θ)]^-1/4^
Primary atom site location: structure-invariant direct methods	Extinction coefficient: 0.0073 (12)

### Special details

**Table:**

Experimental. Empirical absorption correction using spherical harmonics, implemented in SCALE3 ABSPACK scaling algorithm. CrysAlisPro (Agilent Technologies)
Geometry. All s.u.'s (except the s.u. in the dihedral angle between two l.s. planes) are estimated using the full covariance matrix. The cell s.u.'s are taken into account individually in the estimation of s.u.'s in distances, angles and torsion angles; correlations between s.u.'s in cell parameters are only used when they are defined by crystal symmetry. An approximate (isotropic) treatment of cell s.u.'s is used for estimating s.u.'s involving l.s. planes.
Refinement. Refinement of *F*^2^ against ALL reflections. The weighted *R*-factor *wR* and goodness of fit *S* are based on *F*^2^, conventional *R*-factors *R* are based on *F*, with *F* set to zero for negative *F*^2^. The threshold expression of *F*^2^ > 2σ(*F*^2^) is used only for calculating *R*-factors(gt) *etc*. and is not relevant to the choice of reflections for refinement. *R*-factors based on *F*^2^ are statistically about twice as large as those based on *F*, and *R*-factors based on ALL data will be even larger.

### Fractional atomic coordinates and isotropic or equivalent isotropic displacement parameters (Å^2^)

**Table:**

	*x*	*y*	*z*	*U*_iso_*/*U*_eq_	
C1	0.4429 (4)	0.9320 (2)	0.06430 (8)	0.0236 (6)	
H1	0.5156	0.9747	0.0877	0.028*	
C2	0.3207 (4)	1.0088 (2)	0.03673 (9)	0.0276 (6)	
H2	0.2198	0.9692	0.0181	0.033*	
C4	0.2646 (4)	1.1235 (2)	0.04810 (10)	0.0328 (7)	
C5	0.0642 (5)	1.1608 (2)	0.03175 (11)	0.0440 (8)	
H5A	0.0334	1.1215	0.0038	0.053*	
H5B	0.0681	1.2425	0.0256	0.053*	
C6	−0.1006 (5)	1.1365 (3)	0.06533 (12)	0.0500 (9)	
H6A	−0.0928	1.1917	0.0897	0.060*	
H6B	−0.2323	1.1447	0.0510	0.060*	
C7	−0.0803 (4)	1.0198 (2)	0.08363 (11)	0.0377 (7)	
H7	−0.1012	0.9598	0.0635	0.045*	
C8	−0.0368 (5)	0.9912 (3)	0.12466 (10)	0.0396 (8)	
C9	0.0201 (4)	0.8728 (3)	0.13688 (10)	0.0382 (7)	
H9	−0.0145	0.8614	0.1686	0.046*	
C10	0.2457 (4)	0.8572 (2)	0.13205 (9)	0.0306 (6)	
H10A	0.2871	0.7920	0.1502	0.037*	
H10B	0.3133	0.9245	0.1440	0.037*	
C11	0.3171 (4)	0.8384 (2)	0.08456 (8)	0.0227 (6)	
H11	0.1959	0.8291	0.0657	0.027*	
C12	0.4457 (4)	0.7345 (2)	0.07835 (8)	0.0243 (6)	
H12	0.5218	0.7194	0.1061	0.029*	
C13	0.5872 (4)	0.7650 (2)	0.04265 (9)	0.0266 (6)	
C15	0.3307 (4)	0.6301 (2)	0.06537 (9)	0.0288 (6)	
H15A	0.2635	0.6437	0.0368	0.035*	
H15B	0.4256	0.5673	0.0612	0.035*	
C16	0.2748 (4)	0.5460 (2)	0.13653 (9)	0.0309 (7)	
H16A	0.3659	0.6011	0.1504	0.037*	
H16B	0.3552	0.4806	0.1271	0.037*	
C17	0.1246 (4)	0.5089 (3)	0.16934 (9)	0.0358 (7)	
H17A	0.1933	0.4746	0.1949	0.043*	
H17B	0.0488	0.5749	0.1800	0.043*	
C18	−0.1058 (4)	0.4730 (2)	0.11077 (9)	0.0327 (7)	
H18A	−0.1952	0.5357	0.1190	0.039*	
H18B	−0.1878	0.4138	0.0967	0.039*	
C19	0.0472 (4)	0.5141 (2)	0.07857 (9)	0.0328 (7)	
H19A	0.1264	0.4495	0.0678	0.039*	
H19B	−0.0213	0.5480	0.0529	0.039*	
C20	−0.1351 (4)	0.3686 (2)	0.18020 (9)	0.0271 (6)	
C21	−0.0596 (5)	0.3360 (2)	0.22117 (9)	0.0383 (7)	
H21	0.0699	0.3598	0.2297	0.046*	
C22	−0.1693 (5)	0.2703 (3)	0.24923 (10)	0.0429 (8)	
H22	−0.1147	0.2493	0.2769	0.051*	
C23	−0.3549 (5)	0.2348 (3)	0.23802 (10)	0.0413 (8)	
C24	−0.4336 (5)	0.2676 (3)	0.19821 (11)	0.0467 (8)	
H24	−0.5644	0.2447	0.1902	0.056*	
C25	−0.3234 (4)	0.3338 (3)	0.16971 (10)	0.0387 (7)	
H25	−0.3798	0.3555	0.1423	0.046*	
C26	0.3438 (5)	1.1828 (3)	0.08780 (12)	0.0526 (10)	
H26A	0.4715	1.1494	0.0964	0.079*	
H26B	0.2485	1.1756	0.1121	0.079*	
H26C	0.3634	1.2625	0.0809	0.079*	
C27	−0.0231 (7)	1.0695 (3)	0.16314 (13)	0.0744 (13)	
H27A	−0.0360	1.1472	0.1528	0.112*	
H27B	0.1062	1.0598	0.1777	0.112*	
H27C	−0.1304	1.0527	0.1841	0.112*	
C28	−0.6231 (7)	0.1128 (6)	0.2554 (2)	0.134 (3)	
H28A	−0.7310	0.1678	0.2527	0.201*	
H28B	−0.6591	0.0564	0.2776	0.201*	
H28C	−0.6019	0.0758	0.2269	0.201*	
N1	0.1814 (3)	0.59719 (17)	0.09797 (7)	0.0254 (5)	
N2	−0.0123 (3)	0.42794 (18)	0.15021 (7)	0.0286 (5)	
O3	0.4135 (3)	1.10270 (15)	0.01501 (7)	0.0388 (5)	
O4	−0.0873 (3)	0.79295 (17)	0.11206 (8)	0.0440 (6)	
H4	−0.0112	0.7400	0.1049	0.066*	
O5	−0.4482 (4)	0.1672 (2)	0.26829 (8)	0.0655 (8)	
O12	0.6957 (3)	0.70592 (16)	0.02200 (7)	0.0392 (5)	
O14	0.5817 (3)	0.87658 (14)	0.03489 (6)	0.0277 (4)	

### Atomic displacement parameters (Å^2^)

**Table:**

	*U*^11^	*U*^22^	*U*^33^	*U*^12^	*U*^13^	*U*^23^
C1	0.0273 (14)	0.0170 (13)	0.0264 (13)	0.0045 (11)	0.0020 (12)	−0.0043 (11)
C2	0.0301 (14)	0.0167 (12)	0.0360 (15)	−0.0029 (12)	−0.0034 (13)	0.0024 (13)
C4	0.0362 (15)	0.0138 (13)	0.0484 (18)	0.0002 (12)	−0.0031 (14)	−0.0001 (13)
C5	0.0467 (18)	0.0231 (15)	0.062 (2)	0.0085 (14)	−0.0062 (17)	0.0091 (15)
C6	0.0412 (18)	0.0364 (18)	0.072 (2)	0.0170 (16)	−0.0093 (18)	0.0051 (18)
C7	0.0255 (14)	0.0313 (16)	0.056 (2)	0.0021 (14)	−0.0035 (15)	−0.0011 (15)
C8	0.0371 (17)	0.0342 (17)	0.0476 (19)	0.0160 (15)	0.0053 (15)	−0.0058 (15)
C9	0.0415 (17)	0.0354 (16)	0.0377 (16)	0.0084 (14)	0.0085 (15)	−0.0036 (15)
C10	0.0373 (15)	0.0254 (15)	0.0292 (15)	0.0072 (13)	0.0056 (13)	0.0000 (13)
C11	0.0273 (13)	0.0154 (12)	0.0254 (13)	0.0033 (11)	−0.0001 (11)	−0.0005 (11)
C12	0.0321 (14)	0.0164 (13)	0.0243 (13)	0.0055 (11)	0.0037 (12)	−0.0002 (11)
C13	0.0318 (14)	0.0178 (13)	0.0301 (15)	0.0059 (12)	0.0005 (13)	−0.0005 (12)
C15	0.0411 (15)	0.0178 (13)	0.0275 (14)	0.0053 (13)	0.0049 (13)	0.0000 (12)
C16	0.0339 (16)	0.0270 (15)	0.0319 (15)	−0.0020 (12)	−0.0040 (13)	0.0092 (13)
C17	0.0423 (17)	0.0355 (16)	0.0294 (15)	−0.0113 (14)	−0.0069 (14)	0.0075 (13)
C18	0.0419 (17)	0.0257 (14)	0.0304 (15)	−0.0056 (13)	−0.0096 (14)	0.0057 (12)
C19	0.0479 (17)	0.0237 (14)	0.0269 (14)	−0.0051 (14)	−0.0052 (14)	0.0025 (12)
C20	0.0375 (15)	0.0154 (12)	0.0283 (14)	0.0028 (11)	−0.0006 (12)	−0.0014 (11)
C21	0.0473 (18)	0.0352 (17)	0.0325 (15)	−0.0144 (15)	−0.0045 (15)	0.0053 (14)
C22	0.059 (2)	0.0404 (18)	0.0295 (15)	−0.0120 (17)	−0.0055 (16)	0.0058 (15)
C23	0.0438 (18)	0.0407 (18)	0.0395 (18)	−0.0049 (15)	0.0043 (15)	0.0094 (15)
C24	0.0338 (16)	0.048 (2)	0.058 (2)	−0.0046 (16)	−0.0044 (16)	0.0198 (17)
C25	0.0367 (16)	0.0369 (17)	0.0425 (17)	0.0059 (15)	−0.0034 (14)	0.0138 (15)
C26	0.056 (2)	0.0249 (16)	0.077 (3)	0.0041 (16)	−0.0127 (19)	−0.0184 (17)
C27	0.103 (3)	0.056 (2)	0.064 (2)	0.039 (2)	−0.001 (2)	−0.022 (2)
C28	0.076 (3)	0.183 (6)	0.143 (5)	−0.071 (4)	−0.035 (3)	0.119 (5)
N1	0.0358 (13)	0.0180 (11)	0.0223 (11)	−0.0021 (10)	−0.0044 (10)	0.0038 (9)
N2	0.0372 (13)	0.0231 (11)	0.0255 (12)	−0.0044 (10)	−0.0039 (11)	0.0039 (10)
O3	0.0443 (11)	0.0162 (9)	0.0558 (13)	−0.0018 (9)	0.0035 (11)	0.0104 (9)
O4	0.0342 (12)	0.0337 (12)	0.0640 (14)	−0.0016 (10)	0.0071 (11)	0.0012 (11)
O5	0.0575 (15)	0.0798 (19)	0.0593 (15)	−0.0293 (15)	−0.0015 (13)	0.0339 (15)
O12	0.0498 (13)	0.0286 (10)	0.0390 (12)	0.0102 (10)	0.0163 (10)	−0.0017 (9)
O14	0.0314 (10)	0.0177 (9)	0.0341 (10)	0.0027 (8)	0.0081 (9)	0.0003 (8)

### Geometric parameters (Å, º)

**Table:**

C1—O14	1.448 (3)	C16—C17	1.483 (4)
C1—C2	1.485 (4)	C16—H16A	0.9900
C1—C11	1.526 (3)	C16—H16B	0.9900
C1—H1	1.0000	C17—N2	1.452 (3)
C2—O3	1.439 (3)	C17—H17A	0.9900
C2—C4	1.457 (4)	C17—H17B	0.9900
C2—H2	1.0000	C18—N2	1.453 (3)
C4—O3	1.437 (4)	C18—C19	1.499 (4)
C4—C26	1.494 (4)	C18—H18A	0.9900
C4—C5	1.500 (4)	C18—H18B	0.9900
C5—C6	1.530 (4)	C19—N1	1.460 (3)
C5—H5A	0.9900	C19—H19A	0.9900
C5—H5B	0.9900	C19—H19B	0.9900
C6—C7	1.502 (4)	C20—C25	1.367 (4)
C6—H6A	0.9900	C20—C21	1.396 (4)
C6—H6B	0.9900	C20—N2	1.416 (3)
C7—C8	1.323 (4)	C21—C22	1.370 (4)
C7—H7	0.9500	C21—H21	0.9500
C8—C27	1.496 (5)	C22—C23	1.358 (4)
C8—C9	1.507 (4)	C22—H22	0.9500
C9—O4	1.410 (4)	C23—O5	1.371 (4)
C9—C10	1.532 (4)	C23—C24	1.374 (4)
C9—H9	1.0000	C24—C25	1.384 (4)
C10—C11	1.534 (4)	C24—H24	0.9500
C10—H10A	0.9900	C25—H25	0.9500
C10—H10B	0.9900	C26—H26A	0.9800
C11—C12	1.520 (3)	C26—H26B	0.9800
C11—H11	1.0000	C26—H26C	0.9800
C12—C13	1.484 (4)	C27—H27A	0.9800
C12—C15	1.515 (4)	C27—H27B	0.9800
C12—H12	1.0000	C27—H27C	0.9800
C13—O12	1.190 (3)	C28—O5	1.396 (5)
C13—O14	1.350 (3)	C28—H28A	0.9800
C15—N1	1.460 (3)	C28—H28B	0.9800
C15—H15A	0.9900	C28—H28C	0.9800
C15—H15B	0.9900	O4—H4	0.8400
C16—N1	1.460 (3)		
			
O14—C1—C2	106.8 (2)	N1—C16—H16A	109.3
O14—C1—C11	105.74 (18)	C17—C16—H16A	109.3
C2—C1—C11	111.8 (2)	N1—C16—H16B	109.3
O14—C1—H1	110.8	C17—C16—H16B	109.3
C2—C1—H1	110.8	H16A—C16—H16B	107.9
C11—C1—H1	110.8	N2—C17—C16	111.0 (2)
O3—C2—C4	59.49 (17)	N2—C17—H17A	109.4
O3—C2—C1	119.8 (2)	C16—C17—H17A	109.4
C4—C2—C1	125.9 (2)	N2—C17—H17B	109.4
O3—C2—H2	113.6	C16—C17—H17B	109.4
C4—C2—H2	113.6	H17A—C17—H17B	108.0
C1—C2—H2	113.6	N2—C18—C19	111.2 (2)
O3—C4—C2	59.63 (17)	N2—C18—H18A	109.4
O3—C4—C26	113.4 (3)	C19—C18—H18A	109.4
C2—C4—C26	122.8 (3)	N2—C18—H18B	109.4
O3—C4—C5	116.3 (2)	C19—C18—H18B	109.4
C2—C4—C5	115.5 (3)	H18A—C18—H18B	108.0
C26—C4—C5	116.4 (3)	N1—C19—C18	112.4 (2)
C4—C5—C6	111.8 (3)	N1—C19—H19A	109.1
C4—C5—H5A	109.3	C18—C19—H19A	109.1
C6—C5—H5A	109.3	N1—C19—H19B	109.1
C4—C5—H5B	109.3	C18—C19—H19B	109.1
C6—C5—H5B	109.3	H19A—C19—H19B	107.9
H5A—C5—H5B	107.9	C25—C20—C21	117.2 (3)
C7—C6—C5	110.8 (3)	C25—C20—N2	122.6 (2)
C7—C6—H6A	109.5	C21—C20—N2	119.9 (2)
C5—C6—H6A	109.5	C22—C21—C20	121.1 (3)
C7—C6—H6B	109.5	C22—C21—H21	119.5
C5—C6—H6B	109.5	C20—C21—H21	119.5
H6A—C6—H6B	108.1	C23—C22—C21	121.0 (3)
C8—C7—C6	127.2 (3)	C23—C22—H22	119.5
C8—C7—H7	116.4	C21—C22—H22	119.5
C6—C7—H7	116.4	C22—C23—O5	115.7 (3)
C7—C8—C27	126.0 (3)	C22—C23—C24	118.9 (3)
C7—C8—C9	121.9 (3)	O5—C23—C24	125.4 (3)
C27—C8—C9	112.0 (3)	C23—C24—C25	120.3 (3)
O4—C9—C8	111.7 (3)	C23—C24—H24	119.8
O4—C9—C10	111.8 (2)	C25—C24—H24	119.8
C8—C9—C10	109.9 (3)	C20—C25—C24	121.4 (3)
O4—C9—H9	107.7	C20—C25—H25	119.3
C8—C9—H9	107.7	C24—C25—H25	119.3
C10—C9—H9	107.7	C4—C26—H26A	109.5
C9—C10—C11	114.6 (2)	C4—C26—H26B	109.5
C9—C10—H10A	108.6	H26A—C26—H26B	109.5
C11—C10—H10A	108.6	C4—C26—H26C	109.5
C9—C10—H10B	108.6	H26A—C26—H26C	109.5
C11—C10—H10B	108.6	H26B—C26—H26C	109.5
H10A—C10—H10B	107.6	C8—C27—H27A	109.5
C12—C11—C1	103.3 (2)	C8—C27—H27B	109.5
C12—C11—C10	114.3 (2)	H27A—C27—H27B	109.5
C1—C11—C10	116.4 (2)	C8—C27—H27C	109.5
C12—C11—H11	107.5	H27A—C27—H27C	109.5
C1—C11—H11	107.5	H27B—C27—H27C	109.5
C10—C11—H11	107.5	O5—C28—H28A	109.5
C13—C12—C15	109.7 (2)	O5—C28—H28B	109.5
C13—C12—C11	104.7 (2)	H28A—C28—H28B	109.5
C15—C12—C11	114.2 (2)	O5—C28—H28C	109.5
C13—C12—H12	109.4	H28A—C28—H28C	109.5
C15—C12—H12	109.4	H28B—C28—H28C	109.5
C11—C12—H12	109.4	C16—N1—C15	111.1 (2)
O12—C13—O14	120.4 (2)	C16—N1—C19	107.7 (2)
O12—C13—C12	129.1 (2)	C15—N1—C19	109.4 (2)
O14—C13—C12	110.5 (2)	C20—N2—C17	116.3 (2)
N1—C15—C12	113.2 (2)	C20—N2—C18	117.5 (2)
N1—C15—H15A	108.9	C17—N2—C18	110.9 (2)
C12—C15—H15A	108.9	C4—O3—C2	60.88 (17)
N1—C15—H15B	108.9	C9—O4—H4	109.5
C12—C15—H15B	108.9	C23—O5—C28	117.9 (3)
H15A—C15—H15B	107.8	C13—O14—C1	111.0 (2)
N1—C16—C17	111.7 (2)		

### Hydrogen-bond geometry (Å, º)

**Table:**

*D*—H···*A*	*D*—H	H···*A*	*D*···*A*	*D*—H···*A*
O4—H4···N1	0.84	2.14	2.977 (4)	170
C2—H2···O12^i^	1.00	2.42	3.225 (4)	137
C5—H5*B*···O3^ii^	0.99	2.45	3.310 (4)	145
C7—H7···O14^iii^	0.95	2.50	3.198 (4)	130
C15—H15*A*···O12^i^	0.99	2.57	3.413 (4)	143
C15—H15*A*···O14^i^	0.99	2.50	3.469 (4)	165

Symmetry codes: (i) *x*−1/2, −*y*+3/2, −*z*; (ii) *x*−1/2, −*y*+5/2, −*z*; (iii) *x*−1, *y*, *z*.
